# Transforming Growth Factor‐β‐Activated Protein 1 (TAK1) Regulates Necroptosis in Age‐Related Hearing Loss

**DOI:** 10.1111/acel.70013

**Published:** 2025-02-28

**Authors:** Hanjing Wang, Yayun Lv, He Zhao, Zhihong Hao, Xiaoyu Zhai, Yan Wang, Jingjing Qiu, Liang Chen, Jiamin Zhou, Limei Cui, Yan Sun

**Affiliations:** ^1^ School of Clinical Medicine Shandong Second Medical University Weifang Shandong People's Republic of China; ^2^ Department of Otorhinolaryngology, Head and Neck Surgery Yantai Yuhuangding Hospital, Qingdao University Yantai Shandong People's Republic of China; ^3^ Shandong Provincial Key Laboratory of Neuroimmune Interaction and Regulation Yantai Shandong People's Republic of China; ^4^ Shandong Provincial Clinical Research Center for Otorhinolaryngologic Diseases Yantai Shandong People's Republic of China; ^5^ Yantai Key Laboratory of Otorhinolaryngologic Diseases Yantai Shandong People's Republic of China; ^6^ The Second School of Clinical Medicine of Binzhou Medical University Yantai Shandong People's Republic of China; ^7^ Department of Otorhinolaryngology, Head and Neck Surgery Yantai Affiliated Hospital of Binzhou Medical University Yantai Shandong People's Republic of China

**Keywords:** age‐related hearing loss, inflammation, necroptosis, presbycusis, TAK1

## Abstract

Inflammation plays an important role in age‐related hearing loss (ARHL). Transforming growth factor‐β‐activated protein 1 (TAK1), a key factor upstream of inflammatory pathways, mediates various cell death pathways, potentially influencing the survival and death of cochlear hair cells. The DBA/2 J mouse model and the HEI‐OC1 cell line were used to investigate the mechanism of TAK1‐mediated inflammation in ARHL. Hematoxylin and eosin staining revealed significant histological damage in the cochlea of 16‐week‐old mice, along with an increase in auditory‐evoked brainstem response thresholds. Concurrently, TAK1 mRNA levels decreased sharply, and necroptosis significantly increased in 16‐week‐old mice, indicating a correlation between TAK1 expression, necroptosis, and hearing loss. We subsequently constructed TAK1 knockdown and overexpression HEI‐OC1 cells for further investigation. TAK1 knockdown in HEI‐OC1 cells significantly activated the necroptotic pathway, characterized by an increase in necroptosis, along with up‐regulation of *RIPK3* and *MLKL*, and down‐regulation of *NF‐κB* and *Caspase 8*. However, TAK1 overexpression successfully prevented necroptosis in HEI‐OC1 cells, leading to decreases in *NF‐κB*, *Caspase 8*, *RIPK3*, and *MLKL*. We further treated TAK1 knockdown cells with necroptosis inhibitors and found that they could reverse the damage caused by TAK1 knockdown in HEI‐OC1 cells. This preliminary study shows that TAK1‐mediated necroptotic pathways play an important role in the pathogenesis of ARHL.

## Graphical abstract

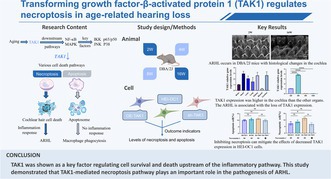

**Abbreviations**
ABR: auditory‐evoked brainstem response
ARHL: age‐related hearing loss
CCK‐8: Cell Counting Kit‐8
D‐gal: D‐galactose
DMEM: Dulbecco's Eagle medium
FADD: FS7‐associated cell surface antigen (Fas)‐associated death domain
HCs: hair cells
H&E: hematoxylin and eosin
HEI‐OC1: house ear institute‐organ of Corti 1
IHCs: inner hair cells
IL‐1β: interleukin‐1β
IL‐6: interleukin‐6
MAPK: mitogen‐activated protein kinase
MLKL: mixed lineage kinase domain like pseudokinase
NF‐κB: nuclear factors κB
OE‐NC: negative transfection of TAK1 overexpressed HEI‐OC1
OE‐TAK1: TAK1 overexpressed HEI‐OC1
OHCs: outer hair cells
PBS: phosphate buffer solution
qRT‐PCR: quantitative real‐time reverse transcription PCR
RIPK1: receptor interacting serine/threonine protein kinase 1
RIPK3: receptor‐interacting protein kinase 3
ROS: reactive oxygen species
SGNs: spiral ganglion neurons
sh‐NC: scramble shRNA‐transfected cells
sh‐TAK1: TAK1 stable knockdown cell line
sh‐TAK1 + Nec‐1: TAK1 stable knockdown cell line + Necrostatin‐1.
SV: stria vascularis
TAK1: transforming growth factor‐β‐activated protein 1
TNFR1: TNF receptor 1
TNF‐α: tumor necrosis factor‐α
TRADD: TNFR‐associated death domain protein

## 1 Introduction

With the global increase in aging populations, age‐related disorders have emerged as a significant social challenge, profoundly impacting the daily lives of the elderly (Bowl and Dawson, 2019). According to the World Health Organization, the global population aged 60 years and older is projected to reach 1.4 billion by 2030, doubling to approximately 2.8 billion by 2050 (Jin et al. 2022). Age‐related hearing loss (ARHL), or presbycusis, is the most prevalent auditory disorder among the elderly, characterized by progressive, bilateral, and symmetrical sensorineural hearing loss (Kociszewska and Vlajkovic 2022). With advancing age, cochlear structures—such as hair cells (HCs), spiral ganglion neurons (SGNs), and the stria vascularis (SV)—undergo varying degrees of damage. The reduction in sound amplification associated with hearing loss primarily results from the degeneration of sensory cells, particularly outer hair cells (OHCs) (Keithley 2020).

The precise mechanisms underlying ARHL remain incompletely understood. It is widely accepted that ARHL arises from a combination of genetic predisposition and cumulative inner ear injuries sustained throughout life (Fu et al. 2023; Wu et al. 2020). Previous research has demonstrated that chronic, low‐grade inflammation is pivotal in the physiological and pathological processes of aging, contributing to the development of various age‐related conditions (Marin‐Aguilar 2018), such as atherothrombosis (Barbu et al. 2022), Alzheimer's disease (Xie 2021), and age‐related macular degeneration (Nashine et al. 2022). However, in‐depth investigations into the role of inflammation in ARHL remain limited. Over the past decades, growing research on inflammatory mechanisms has led otolaryngologists to hypothesize that inflammation in the cochlea, particularly involving the blood‐labyrinth barrier, contributes to ARHL (Watson et al. 2017). Inflammatory markers, including interleukin (IL)‐6, IL‐1β, and tumor necrosis factor (TNF)‐α, have been observed to exhibit elevated expression levels in aging cochleae (Wang and Puel 2020). Our prior findings provide evidence that a TNF‐α‐mediated inflammatory pathway contributes to the pathogenesis of ARHL (Wu et al. 2022).

Transforming growth factor‐β‐activated protein 1 (TAK1), a member of the mitogen‐activated protein kinase (MAPK) family, is implicated in the regulation of diverse physiological and pathological processes (Xu and Lei 2020). Recent studies have revealed that TAK1 is activated by diverse extracellular stimuli, functioning as a pivotal regulator of cell death and a critical mediator of both innate and adaptive immunity (Mihaly 2014). TAK1 is an essential downstream effector in nuclear factor κB (NF‐κB) signaling pathways, activated by various pattern recognition and cytokine receptors (Sakurai 2012). The previous study has demonstrated that TAK1‐mediated inflammatory responses contribute to multiple forms of cell death (Malireddi, Kesavardhana, and Kanneganti 2019). Deletion or knockdown of TAK1 redirects NF‐κB signaling from survival pathways to cell death pathways, including apoptosis, pyroptosis, and necroptosis (Malireddi et al. 2020). Apoptosis and pyroptosis are induced by the activation of caspase‐8 (CASP8), whereas inhibition of CASP8 triggers necroptosis, regulated by receptor‐interacting serine/threonine‐protein kinase 3 (RIPK3) and mixed‐lineage kinase domain‐like pseudokinase (MLKL) (Fritsch et al. 2019; Mandal et al. 2020). TAK1 plays a critical role in regulating cell survival, cell death, and inflammation in various diseases, including cancer (Podder et al. 2019), rheumatoid arthritis (Anderton, Wicks, and Silke 2020), and liver cirrhosis (Tan et al. 2020). However, the role of TAK1 in regulating ARHL‐related mechanisms remains poorly understood.

Building upon the above findings, we hypothesize that TAK1 plays a pivotal role in the pathogenesis of ARHL. In this study, we observed a reduction in TAK1 expression correlated with cochlear structural damage in DBA/2 J mice, a widely used model for ARHL. A similar observation was made in an in vitro cell model, where reduced TAK1 expression was linked to necroptosis. This study aims to elucidate the role of TAK1 in ARHL pathogenesis by modulating its expression. Our findings uncover a novel mechanism underlying ARHL, offering valuable insights for clinical treatment and pharmacological research.

## 2 Methods

### 2.1 Animals

Male DBA/2 J mice were procured from the Model Animal Research Center (Shanghai Laboratory Animal Center, China) and raised in a specific pathogen‐free animal facility at the Yantai Yuhuangding Hospital Affiliated with Qingdao University. All animal‐related experimental procedures adhered to the guidelines outlined in the Guide for the Care and Use of Laboratory Animals. This study received ethical approval from the Animal Use and Care Committee of Yantai Yuhuangding Hospital Affiliated with Qingdao University (Approval No. 2022–377). All experiments included at least six mice per group, and each experiment was repeated at least three times. Quantification of OHC numbers, SGN density, and SV width was conducted in the middle turn of the mouse cochlea. Three regions were randomly selected and photographed in each cochlea, and images from all mice in different groups were pooled for statistical analysis.

### 2.2 Auditory‐Evoked Brainstem Response (ABR)

Male DBA/2 J mice were categorized into four age groups: 2, 4, 8, and 16 weeks. ABR thresholds were measured using a computer‐aided evoked potential system (Intelligent Hearing System, USA). Headphones were positioned over the external acoustic meatus to deliver pure‐tone stimuli to the ear. The inner ears of the mice were stimulated briefly with short pure tones at various frequencies (8, 16, and 32 kHz) as well as a broadband click stimulus via headphones. The sound intensity began at 20 dB and was gradually raised. The ABR threshold for each mouse was determined as the minimum sound intensity required to elicit a discernible wave III response. Hearing levels were classified as follows: normal hearing (≤ 25 dB), mild hearing loss (26–40 dB), moderate hearing loss (41–60 dB), severe hearing loss (61–80 dB), profound or total hearing loss (≥ 81 dB).

### 2.3 Hematoxylin and Eosin (HE) Staining

DBA/2 J mice were anesthetized via intraperitoneal injection of 1% sodium pentobarbital at 40 mg/kg. Following systemic perfusion with phosphate‐buffered saline (PBS) and fixation using 4% paraformaldehyde, the cochleae were harvested. The harvested cochleae were further fixed in 4% paraformaldehyde for 24 h, decalcified in 10% ethylenediaminetetraacetic acid (EDTA) for 3–5 days, embedded in paraffin, sectioned at 5 μm thickness using a Rotary Microtome (Leica, Germany), and stained with hematoxylin and eosin (H&E) using a staining kit (G1120, Solarbio, China) according to the manufacturer's instructions. The HCs, SGNs, and SV in the scala media were examined under a microscope (Olympus, Japan). The number of OHCs rows, SGNs density, and SV width in the middle turn of cochleae were recorded.

### 2.4 Scanning Electron Microscope (SEM)

The anatomical fixation and decalcification of the cochleae were conducted following previously established protocols. The base, middle, and apex turns of the cochlea were dissected under a microscope. The cochleae were trimmed to expose the basilar membrane, and the capping membrane was removed. The trimmed basilar membrane was fixed in 1% osmium tetroxide for 1.5 h and rinsed with 0.1 M PBS.

The samples were sequentially dehydrated in graded ethanol concentrations. The membranes were then dried in an anhydrous ethanol and hexamethyldisilazane mixture (1:1) followed by 100% hexamethyldisilazane. Finally, the basilar membrane was amounted onto the electron microscope carrier stage. The HCs were observed using a scanning electron microscope (SEM; ZEISS Sigma 300/500 Field Emission Scanning Electron Microscope).

### 2.5 Cell Culture

Mouse cochlear hair cell line HEI‐OC1 cells and HEK‐293 T cells were obtained from American Type Culture Collection (ATCC, USA) and cultured in high‐glucose Dulbecco's Modified Eagle Medium (DMEM; Gibco, USA) supplemented with 10% fetal bovine serum (FBS; Gibco, USA). HEK‐293 T cells were incubated at 37°C in a 5% CO_2_ incubator, while HEI‐OC1 cells were maintained at 33°C in a 10% CO_2_ incubator.

### 2.6 Immunofluorescence Stain

The cochleae of 2‐week‐old mice were isolated, fixed with 4% paraformaldehyde, decalcified with 10% EDTA, paraffin‐embedded, and sectioned as previously described. The sections were then baked, dewaxed, and prepared for immunofluorescence. For immunofluorescence staining of cultured cells, the cells were seeded onto a cover glass in a culture dish at an appropriate density. Cells on the cover glass were fixed in 4% paraformaldehyde after culturing. For immunofluorescence staining, the sections were infiltrated in PBS containing 0.1% Triton X‐100, blocked with 3% bovine serum albumin (BSA), and then incubated overnight with rabbit TAK1 polyclonal antibody (1:500, 67,707‐1‐lg, Proteintech, China) or TUJ1 polyclonal antibody (1:300, ab18207, Abcam, UK). The following day, the tissue sections or cover glasses with cultured cells were incubated in Goat anti‐Rabbit IgG Alexa Fluor 594 (1:200, AS074, ABclonal, China) or Goat anti‐Rabbit IgG Alexa Fluor 488 (1:500, AS053, ABclonal, China). Finally, the tissue sections or cover glasses with cultured cells were mounted with an anti‐fluorescence quenching agent containing DAPI (P0131, Beyotime, China) and photographed under a fluorescence microscope (Zeiss, Germany).

### 2.7 TAK1 Knockdown and Overexpression Cells Construction

Lentiviral pLKO.1 shRNA expression systems were used to generate a TAK1 stable knockdown cell line (sh‐TAK1) and a scramble control (sh‐NC). Briefly, 2 μg of synthesized plasmids (psPAX2, pMD2.G, sh‐TAK1/sh‐scramble; Sangon Biotech, China) in 200 μL of jetPRIME® buffer (Polyplus Transferion, France) were mixed with 4 μL of jetPRIME® reagent and added to HEK‐293 T cells. After 48 and 72 h, the viral supernatant was collected and added to HEI‐OC1 cells, along with 1.25 μg/mL polybrene (Yeasen, Shanghai, China) to enhance infection efficiency. The infected sh‐TAK1 and sh‐NC HEI‐OC1 cells were selected using 2 μg/mL puromycin (Beyotime). The sh‐TAK1 sequence was: CCGGCGCCCTTCAATGGAGGAAATTCTCGAGAATTTCCTCCATTGAAGGGCGTTTTTG.

To construct TAK1 overexpressed HEI‐OC1 (OE‐TAK1), 200 μL of jetPRIME® buffer containing 2 μg of DNA (TAK1‐pcDNA3.1 or pcDNA3.1 plasmid) (Sangon Biotech) was mixed with 4 μL of jetPRIME® reagent, incubated at room temperature for 10 min, and transfected into the prepared HEI‐OC1 cells.

### 2.8 Quantitative Real‐Time Reverse Transcription PCR (qRT‐PCR)

SparkZol reagent (Sparkjade, China) was used to extract RNA from the whole cochlea or cells. A SPARKscript II RT Plus Kit (AG0304‐A, Sparkjade) was used for reverse transcription, according to the manufacturer's guidelines. SYBR Green qPCR Mix (AH0101‐A, Sparkjade) was then used for quantitative reverse transcription polymerase chain reaction (qRT‐PCR) detection in a StepOnePlus fluorescence quantitative PCR cycler (Applied Biosystems, USA), following the product's instructions. Finally, mRNA expression was normalized to the β‐actin expression level, and a $2^{-\Delta \Delta C_{t}}$ method was used for calculation. Primer information for this experiment is provided in Table 1.

**TABLE 1:** Primer information for all relevant genes selected for qRT‐PCR.

ID	Sequence
TAK1‐F	5'‐CGGATGAGCCGTTACAGTATC‐3'
TAK1‐R	5'‐CGGATGAGCCGTTACAGTATC‐3'
NF‐κB‐F	5'‐GCTGCCAAAGAAGGACACGACA −3'
NF‐κB‐R	5'‐GGCAGGCTATTGCTCATCACAG −3'
Caspase‐8‐F	5'‐TGCTTGGACTACATCCCACAC‐3'
Caspase‐8‐R	5'‐TGCAGTCTAGGAAGTTGACCA‐3'
RIPK3‐F	5'‐TCTGTCAAGTTATGGCCTACTGG‐3'
RIPK3‐R	5'‐TCTGTCAAGTTATGGCCTACTGG‐3'
MLKL‐F	5'‐AATTGTACTCTGGGAAATTGCCA‐3'
MLKL‐R	5'‐TCTCCAAGATTCCGTCCACAG‐3'
P16‐F	5'‐AACTCTTTCGGTCGTACCCC‐3'
P16‐R	5'‐GCGTGCTTGAGCTGAAGCTA‐3'
P21‐F	5'‐CCTGGTGATGTCCGACCTG‐3'
P21‐R	5'‐CCTGGTGATGTCCGACCTG‐3'
Actin‐F	5'‐GGCTGTATTCCCCTCCATCG‐3'
Actin‐R	5'‐CCAGTTGGTAACAATGCCATGT‐3'

### 2.9 Western Blotting (WB)

Protein concentration in the lysate was determined using a BCA Protein Quantitative Kit (EC0001‐A, Sparkjade), according to the manufacturer's instructions. Protein aliquots were loaded onto a 12% sodium dodecyl‐sulfate polyacrylamide gel electrophoresis, electrophoresed, and transferred onto a nitrocellulose membrane (Millipore, Billerica, MA, USA). The membrane was blocked with 5% BSA and incubated with TAK1 polyclonal antibody (1:1000, 67,707‐1‐lg, Proteintech), RIP3 polyclonal antibody (1:1000, 17,563‐1‐AP, Proteintech), or MLKL monoclonal antibody (1:2000, 66,675‐1‐Ig, Proteintech) at 4°C overnight. After incubating with the corresponding secondary antibody (1:2500, B900210, Proteintech), probed proteins were visualized using enhanced chemiluminescent substrate (Sparkjade) and imaged using a chemiluminescence imaging system (ChemiScope 6200 Touch; Clinx Science, China). Protein expression levels were quantified using ImageJ software (V1.8.0.112).

### 2.10 Cellular Viability Assay

Cells were incubated in a well of a 96‐well culture plate containing 100 μL of complete DMEM medium for more than 6 h and treated with different concentrations of D‐gal (SG8010, Solarbio, China) (0, 10, 20, 30, 40, and 50 mg/mL) or Necrostatin‐1 (HY‐15760, MedChemExpress) (0, 20, 40, 60, 80, and 100 μM). After 24, 48, and 72 h of treatment, cellular proliferation was assessed using a Cell Counting Kit‐8 (CCK‐8) (CT0001‐B, Sparkjade) reagent, following the manufacturer's instructions.

### 2.11 Reactive Oxygen Species (ROS) Detection

Cells were seeded in 6‐well culture plates at a density of 1 × 10^6^ cells/mL, pre‐incubated in a cell incubator for 6 h, and treated with 40 mg/mL D‐gal for 48 h. Reactive oxygen species (ROS) activity was detected using an ROS assay kit (S0033S, Beyotime, Jiangsu, China), following the manufacturer's instructions. Cells were photographed and analyzed under a fluorescence microscope (Zeiss, Germany).

### 2.12 Apoptosis and Necroptosis Assay

Cells were digested with trypsin‐free pancreatic enzymes and collected. Apoptosis and necroptosis rates were assayed by a flow cytometer method, according to the protocol of the Annexin V‐fluorescein isothiocyanate (FITC)/propidium iodide (PI) Apoptosis Detection Kit (Vazyme Biotech, Nanjing, China). Briefly, after washing the cells, 1× binding buffer, Annexin V‐FITC, and PI staining solution were added and incubated at room temperature in the dark for 10 min. After the addition of 400 μL of 1× binding buffer, the flow cytometer was performed using the MoFlo XDP high‐speed sorting type flow cytometer (Beckman Coulter, CA, USA).

### 2.13 Enzyme‐Linked Immunosorbent Assay (ELISA) for Interleukin 1β (IL‐1β) in Mice

The adherent cells were washed with pre‐chilled PBS, digested with trypsin, and collected by centrifugation at 1000 × g for 5 min. 1 × 10^6^ cells were washed 3 times with pre‐chilled PBS, resuspended with 200 μL PBS, and protease inhibitors were added at a ratio of 1:100. The cells were fragmented by sonication. The extracts were centrifuged at 4°C, 1500 × g for 10 min, and the supernatant was collected. The IL‐1β concentration of each group of cells was measured using an ELISA kit (JL18442, Jonlnbio, China) according to the manufacturer's instructions.

### 2.14 Lactate Dehydrogenase (LDH) Assay

Cells were seeded at a density of 6.25 × 10^4^ cells/mL in 96‐well plates with 50 μL per well. Adherent cells were cultured overnight, and 50 μL of medium containing the drug was added after changing the medium. The cells were cultured in a cell culture incubator at 33°C and 10% CO_2_ for 48 h. The absorbance at 490 nm of each group of cells was measured using the LDH Cytotoxicity Assay Kit (MA0649, Meilunbio, China), according to the manufacturer's instructions. The absorbance was positively correlated with the amount of LDH.

### 2.15 Statistical Analysis

The differences among three or more groups were tested with one‐way ANOVA. Data between the two groups were analyzed using an unpaired two‐tailed Student's t‐test. Results were considered statistically significant if *p* < 0.05. Error bars represent the standard deviations from the mean of three independent assays. All experiments were repeated at least three times.

## 3 Results

### 3.1 ARHL Accompanied by Histological Cochlear Damage in Older DBA/2 J Mice

**FIGURE 1:**
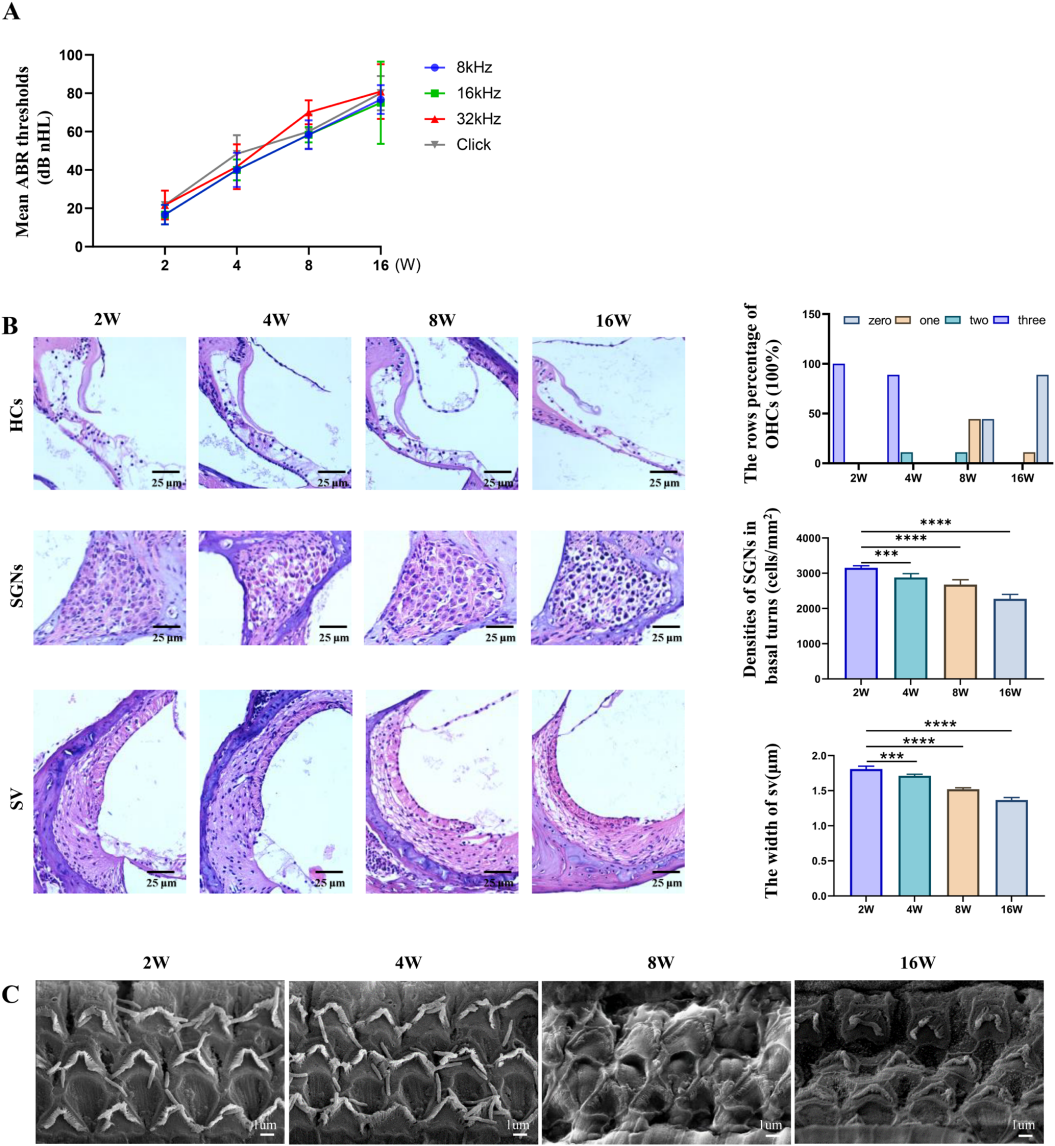
Auditory‐evoked brainstem response thresholds, histological and morphological changes of the cochlea at various ages (2–16 weeks) of DBA/2 J mice. (A) Auditory‐evoked brainstem response thresholds were analyzed in DBA/2 J mice of various ages at 8 (blue), 16 (green), and 32 (red) kHz, as well as with click auditory (gray) stimuli. (B) The morphological characteristics of hematoxylin and eosin (H&E)‐stained (×200) mouse cochlear outer hair cells (OHCs), spiral ganglion neurons (SGNs), and vascular striae (SVs) in different ages of DBA/2 J mice. The quantitative statistical plots of the percentage of OHCs in each row, SGN density (cells/mm^2^), and SV width (μm) were shown on the right. (C) The morphological changes of middle turn stereocilia by SEM in different ages of DBA/2 J mice. (*N* = 6, ***p < 0.001, ****p < 0.0001).

ABR levels were measured in DBA/2 J mice aged 2, 4, 8, and 16 weeks to observe ARHL. The mean ABR threshold was essentially normal in 2‐week‐old mice. With increasing age, the mean ABR thresholds at each frequency were elevated. Severe deafness was eventually observed in the 16‐week‐old mice (Figure 1A). Hearing damage in the mice occurred early and was progressively increased in severity. Alterations in the middle turn of cochlear structure were examined by H&E staining of tissue sections in each age group. The results showed a progressive loss of HCs, a degeneration of SGNs, and a narrowing of SV width in the cochlea of the DBA/2 J mice with increasing age. The OHCs and IHCs of the 2‐week‐old mice were intact. However, the number of OHCs began to decrease after 4 weeks of age. SGN density and SV width gradually decreased from 8 weeks of age, reaching their lowest levels at 16 weeks of age (Figure 1B) (*p* < 0.01). Morphological changes in the middle turn stereocilia were observed by SEM in each age group of DBA/2 J mice. Consistent with the loss of HCs, stereociliary disarrangement was already apparent at 8 weeks of DBA/2 J mice, and the stereocilia were completely lost in 16 weeks (Figure 1C). These data indicate that ARHL occurs in DBA/2 J mice, with histological changes in the cochlea observable at 4 weeks, establishing them as a suitable model for ARHL.

### 3.2 Decreased Expression of TAK1 in Elder DBA/2 J Mice

**FIGURE 2:**
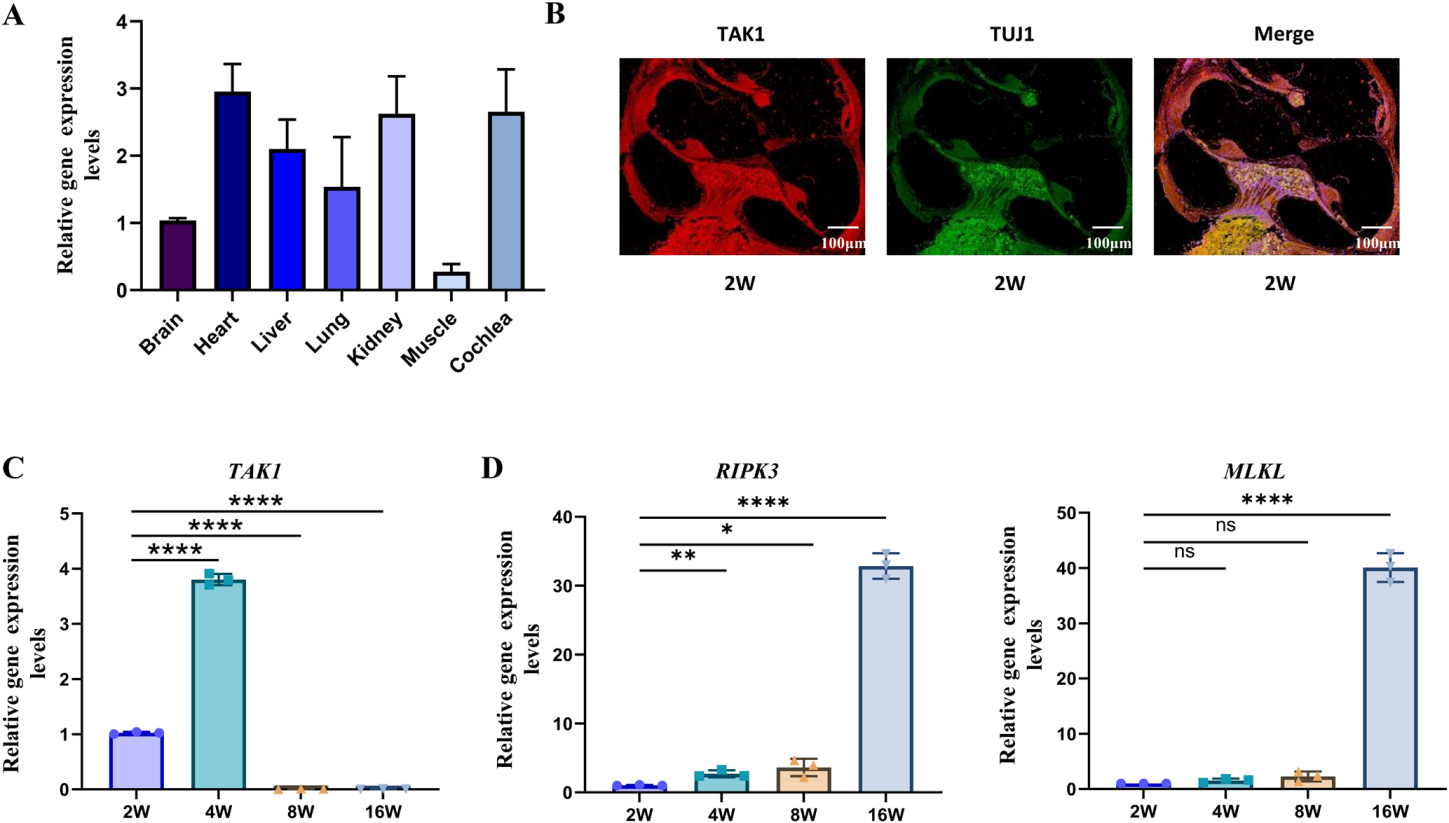
TAK1 expression in DBA/2 J mice. (A) Relative TAK1 gene expression levels were examined by qRT‐PCR in different tissues of DBA/2 J mice at 2 weeks of age. (B) Localization of TAK1 was determined by immunofluorescence staining (TUJ1 was used as an SGNs marker). (C) The relative mRNA levels of TAK1 were detected by qRT‐PCR in the cochlear tissue of DBA/2 J mice. (D) The relative mRNA levels of RIPK3 and MLKL in cochlear tissues of DBA/2 J mice were detected by qRT‐PCR. Each data point was triplicated (*p < 0.05, **p < 0.01, ****p < 0.0001; ns, no significance).

To investigate the impact of TAK1 on ARHL, a preliminary analysis of TAK1 expression levels in different organs of the 2‐week‐old DBA/2 J mice was conducted using qRT‐PCR. Interestingly, TAK1 expression was higher in the cochlea than in the heart and kidneys. This suggests that TAK1 may play a significant role in cochlear function (Figure 2A). Immunofluorescence staining of the cochlear tissue of 2‐week‐old mice showed that TAK1 was expressed on HCs, SGNs, and the SV (Figure 2B). TAK1 was observed to be expressed in both hair cells and spiral ganglion neurons. TAK1 expression levels were subsequently measured by qRT‐PCR in the cochleae of mice of each age group. The results showed that TAK1 levels were detectable in the younger mice (2 weeks old), peaked at 4 weeks of age, then declined and became undetectable from 8 weeks of age onward (Figure 2C) (*p* < 0.01). Moreover, the expressions of necroptosis markers *RIPK3* and *MLKL* increased with age (Figure 2D) (*p* < 0.01). These results indicate that the hearing loss in DBA/2 J mice is associated with necrotic apoptosis, which may be related to the loss of TAK1 expression.

### 3.3 TAK1 Expression Decreased in D‐Gal‐Induced Aged HEI‐OC1 Cells

**FIGURE 3:**
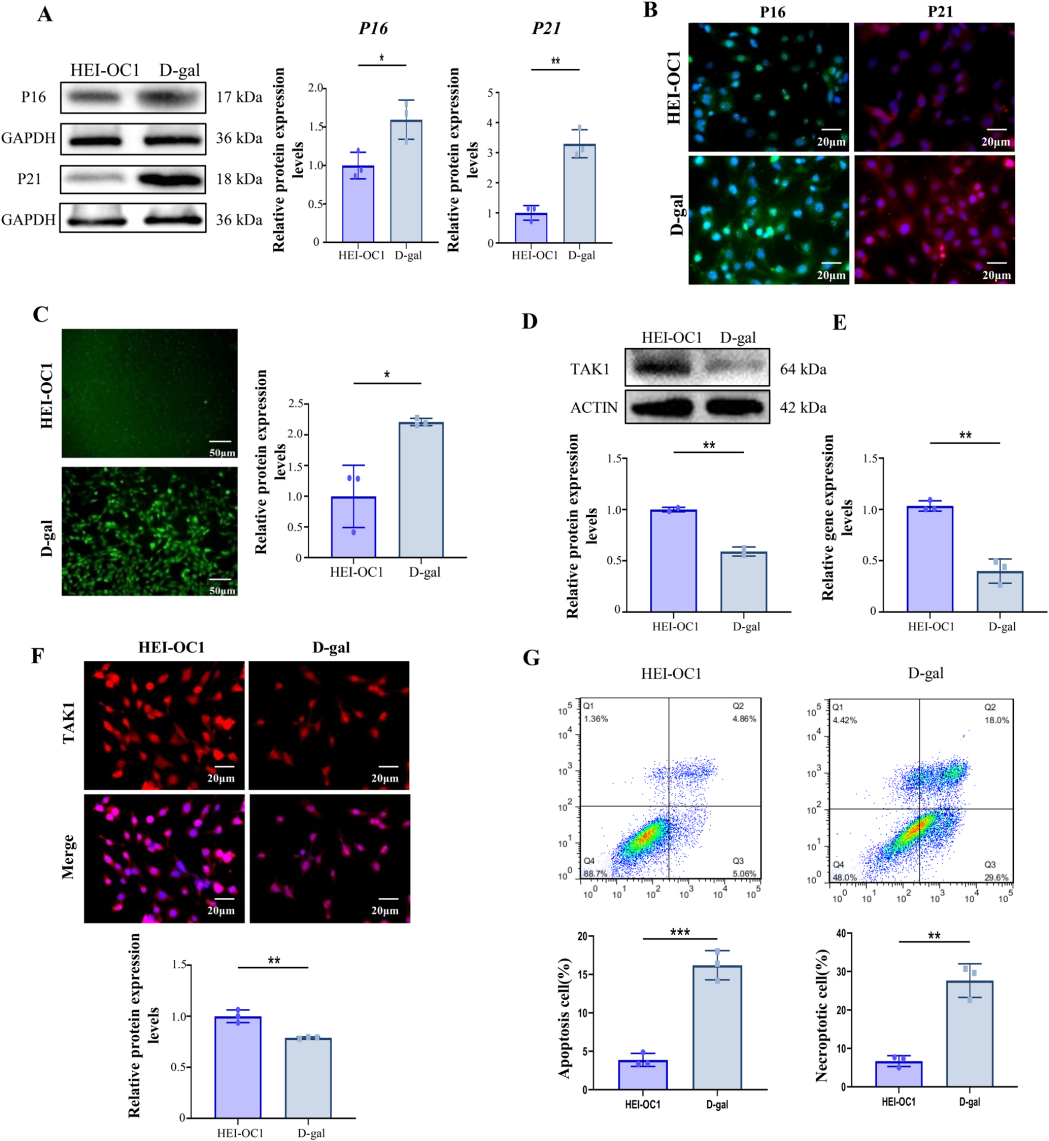
TAK1 decrease and necroptosis increase in D‐gal‐induced aging HEI‐OC1 cells. (A) Relative protein levels of P16 and P21 were detected by western blotting. (B) Immunofluorescence staining of P16 and P21. (C) Reactive oxygen species staining in D‐gal‐treated cells and control cells. (D) The relative protein levels of TAK1 were analyzed by western blotting in D‐gal‐treated cells and control cells. (E) The relative mRNA levels of TAK1 were detected by qRT‐PCR in D‐gal‐treated cells and control cells. (F) The relative protein expression of TAK1 was detected by immunofluorescence staining in D‐gal‐treated cells and control cells. (G) Cellular necroptosis was analyzed by flow cytometer assay. Each data point was repeated in three independent assays (*p < 0.05, **p < 0.01, ***p < 0.001).

To further investigate the relationship between decreased TAK1 and HCs aging, we established a D‐gal‐induced aging cellular model in HEI‐OC1 cells in vitro. First, HEI‐OC1 cells were treated with 0, 10, 20, 30, 40, and 50 mg/mL D‐gal for 24, 48, and 72 h. Cellular viability was then assessed using the CCK‐8 assay. The results showed that cellular viability significantly decreased when cells were treated with 40 mg/mL D‐gal for 48 h compared with negative controls (0 mg/mL D‐gal) (Figure S1A) (*p* < 0.01). Next, we analyzed the expression of aging marker genes, P16 and P21, by WB, immunofluorescence staining, and qRT‐PCR (Figure 3A,B, Figure S1B,C) (*p* < 0.01). Results showed that both mRNA and protein levels of P16 and P21 were significantly increased in 40 mg/mL D‐gal‐treated HEI‐OC1 cells, indicating that the cells had become senescent. Since overproduction of ROS is a key mechanism of ARHL, we used ROS levels as an indicator of aging cells. Intracellular ROS levels were analyzed by kit. Notably, ROS‐stained cells in the 40 mg/mL D‐gal‐treated group were significantly increased compared to the control group, suggesting successful construction of the aged HEI‐OC1 cell model (Figure 3C) (*p* < 0.05). To examine TAK1 profiles in aging cells and cell damage, we performed WB, qRT‐PCR, and immunofluorescence staining. The results revealed that TAK1 expression levels, both gene and protein, were significantly decreased in the D‐gal‐induced group compared with the control group (Figure 3D–F) (*p* < 0.01). In addition, flow cytometer analysis indicated that apoptosis and necroptosis levels were notably increased in the D‐gal‐induced group compared to the control group (Figure 3G) (*p* < 0.01). These data further confirm that TAK1 expression was decreased in D‐gal‐induced aged HEI‐OC1 cells, and this decrease was correlated with an increase in cell necroptosis.

### 3.4 TAK1 Knockdown Leads to HEI‐OC1 Cell Death Through Necroptosis

**FIGURE 4:**
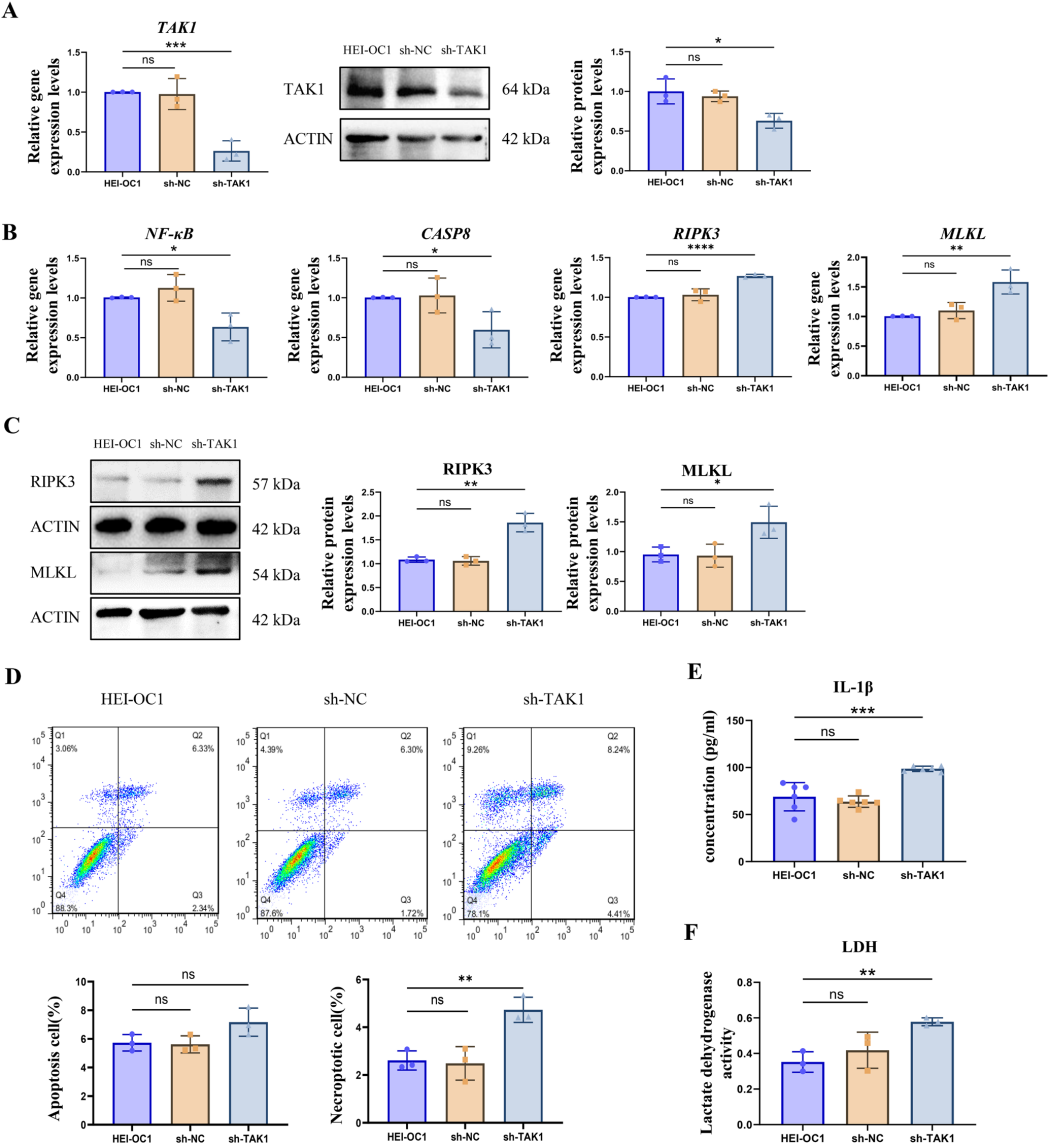
TAK1 knockdown leads to HEI‐OC1 cell death through necroptosis. (A) The relative mRNA and protein levels of TAK1. (B) The relative mRNA expression levels of *NF‐κB, CASP8*, *RIPK3*, and *MLKL* were detected by qRT‐PCR. (C) The relative protein levels of RIPK3 and MLKL were analyzed by western blotting and quantified by image J. (D) Apoptosis and necroptosis were analyzed by flow cytometry. (E) The relative expression levels of IL‐1βwere analyzed by ELISA. (F) The amount of LDH was detected. Each data point was repeated in three independent assays (*p < 0.05, **p < 0.01, ***p < 0.001, ****p < 0.0001; ns, no significance).

TAK1 can mediate several cell death pathways, including apoptosis, pyroptosis, and necroptosis. To further investigate the specific mechanism underlying decreased TAK1 expression in aging cochlear and HEI‐OC1 cells, we constructed a TAK1 knockdown HEI‐OC1 cell using stable transfection. The mRNA levels of TAK1 were detected by qRT‐PCR, and protein levels were analyzed by WB in sh‐TAK1, sh‐NC, and non‐transfected cells (HEI‐OC1). The results showed that the mRNA and protein expression levels of TAK1 were significantly down‐regulated in the sh‐TAK1 group (Figure 4A) (*p* < 0.01). Next, we examined cell death pathways by qRT‐PCR in the sh‐TAK1 cells. The results indicated that the mRNA levels of *NF‐κB* and *CASP8* were significantly decreased in the sh‐TAK1 cells compared to wild‐type and negative transfection controls. However, the mRNA levels of *RIPK3* and *MLKL* were upregulated in the sh‐TAK1 cells (Figure 4B) (*p* < 0.01). The expression levels of *RIPK3* and *MLKL* were then reconfirmed at the protein level by WB in the sh‐TAK1 cells (Figure 4C) (*p* < 0.01). A flow cytometer was used to assess apoptosis and necroptosis levels in the sh‐TAK1 cells. The results showed that, compared to normal controls, there was no significant change in the percentage of apoptosis after sh‐TAK1 treatment; however, necroptosis was significantly increased in the sh‐TAK1 cells (Figure 4D) (*p* < 0.01). IL‐1β and LDH are two markers of necroptosis, and we further examined their expression changes. ELISA data showed that the concentration of IL‐1β was significantly increased in the sh‐TAK1 group (Figure 4E) (*p* < 0.01). The LDH detection experiment showed that the amount of LDH released in the sh‐TAK1 group was significantly higher than in the control group (Figure 4F) (*p* < 0.01). These data suggest that the low TAK1 expression leads to activation of the necroptosis pathway in HEI‐OC1 cells.

### 3.5 TAK1 Overexpression Prevents Cellular Necroptosis in HEI‐OC1 Cells

**FIGURE 5:**
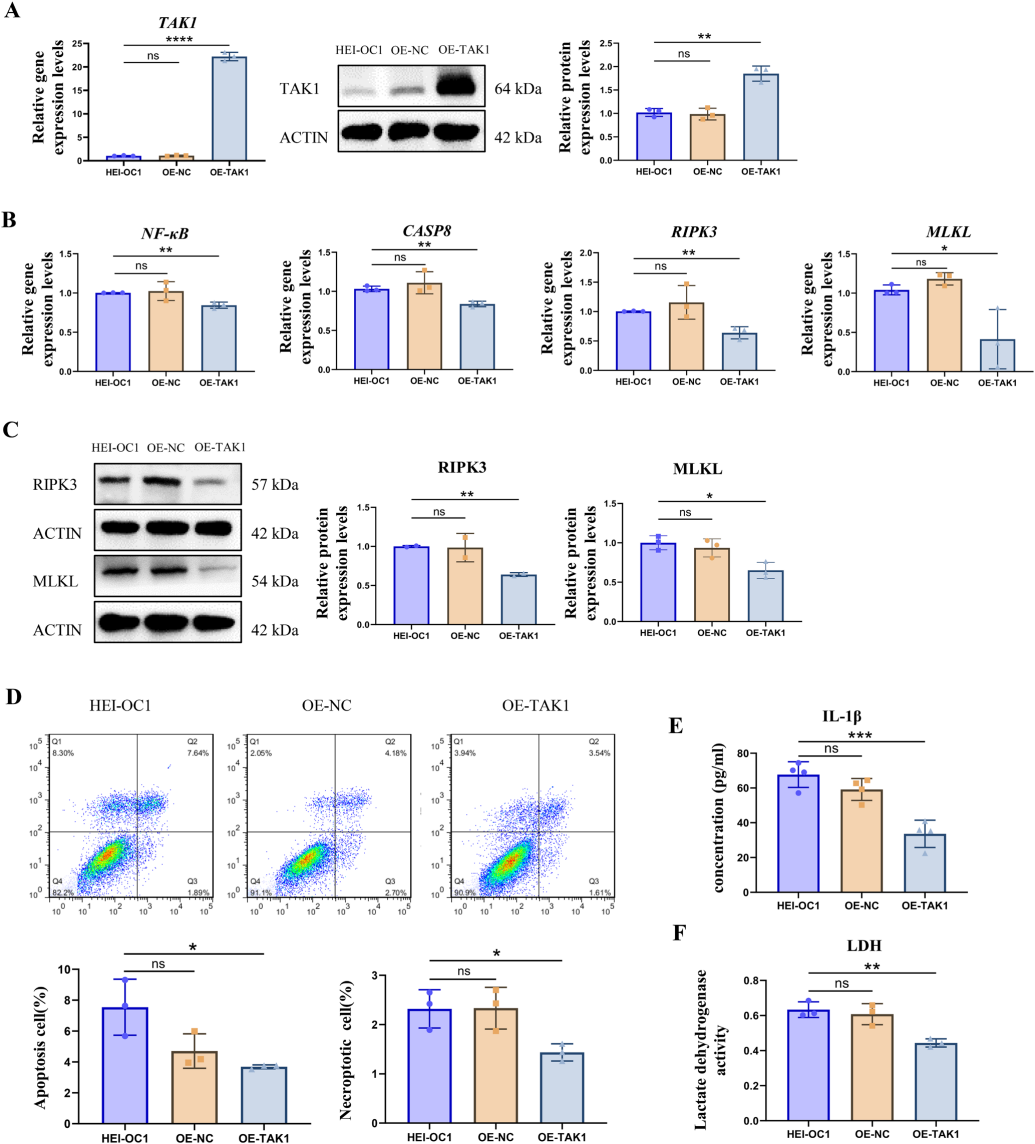
TAK1 overexpression prevents cellular necroptosis in HEI‐OC1 cells. (A) The relative mRNA and protein expression levels of TAK1 in OE‐TAK1 cells. (B) The relative mRNA expression levels of NF‐κB, CASP8, RIPK3, and MLKL were detected by qRT‐PCR. (C) The protein expression levels of RIPK3 and MLKL were analyzed by western blotting. Quantification was performed by ImageJ software. (D) Apoptosis and necroptosis were analyzed by flow cytometry. (E) The relative expression levels of IL‐1βwere analyzed by ELISA. (F) The amount of LDH was detected. Each data point was repeated in three independent assays (*p < 0.05, **p < 0.01, ***p < 0.001, ****p < 0.0001; ns, no significance).

TAK1 can activate NF‐κB signaling to regulate the transcription of genes involved in balanced cell death and survival. To investigate the survival effects of TAK1 in HEI‐OC1 cells, we constructed a TAK1 overexpression HEI‐OC1 cell line. After confirming the higher gene and protein expression levels of OE‐TAK1 by qRT‐PCR and WB (Figure 5A) (*p* < 0.01), the mRNA and protein expression levels of necroptosis‐related factors, including *NF‐κB*, *CASP8*, *RIPK3*, and *MLKL*, were examined in OE‐TAK1 cells. The results indicated that the mRNA levels of *NF‐κB*, *CASP8*, *RIPK3*, and *MLKL* were significantly decreased in the OE‐TAK1 group (Figure 5B) (*p* < 0.05). The reductions in *RIPK3* and *MLKL* were also confirmed by WB (Figure 5C) (*p* < 0.05). Flow cytometer data showed that both apoptosis and necroptosis were reduced in the OE‐TAK1 group (Figure 5D) (*p* < 0.05). The ELISA data showed that the cellular IL‐1β concentration in the OE‐TAK1 group was significantly lower than in the HEI‐OC1 group (Figure 5E) (*p* < 0.01). The results of the LDH cytotoxicity assay showed that the amount of LDH in the OE‐TAK1 group was significantly lower than in the HEI‐OC1 group (Figure 5F) (*p* < 0.01). These data reinforce the idea that TAK1 is a crucial factor in regulating cellular necroptosis in HEI‐OC1 cells.

### 3.6 Inhibiting Necroptosis Can Rescue Cell Damage Induced by the Deficiency of TAK1

**FIGURE 6:**
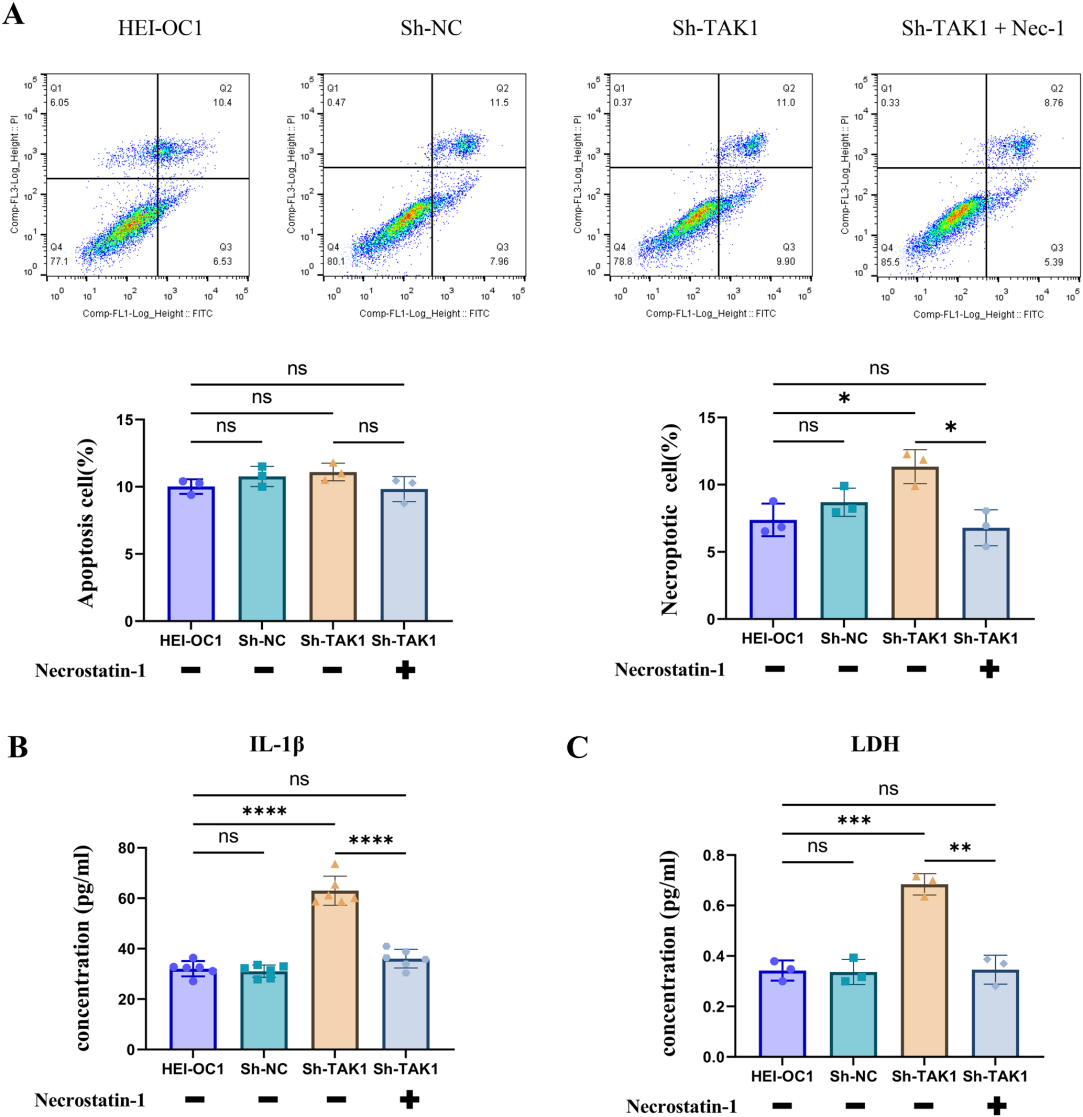
Inhibiting necroptosis can rescue cell damage induced by TAK1 deficiency. (A) Apoptosis and necroptosis were analyzed by flow cytometry. (B) The IL‐1βexpression levels were analyzed by ELISA. (C) The amount of LDH was detected. Each data point was repeated in three independent assays (*p < 0.05, **p < 0.01, ***p < 0.001, ****p < 0.0001; ns, no significance).

All results showed that decreased TAK1 expression leads to the activation of necroptosis in DBA/2 J mice and HEI‐OC1 cells. To further investigate the role of TAK1 in ARHL, primarily through the regulation of cell necroptosis, we used Necrostatin‐1 to suppress necroptosis and subsequently assessed the impact of TAK1 knockdown on HEI‐OC1 cells. HEI‐OC1 cells were treated with 0, 20, 40, 60, 80, and 100 μM Necrostatin‐1 for 24, 48, and 72 h. The results showed that the treatment of HEI‐OC1 cells with 20 μM Necrostatin‐1 for 48 h was optimal (Figure S2A). Flow cytometer data indicated that after the addition of Necrostatin‐1, there was no significant difference in the proportion of apoptosis and necroptosis between the sh‐TAK1 group and the HEI‐OC1 group (Figure 6A). Consistently, after the addition of Necrostatin‐1, the expression levels of IL‐1β and LDH in the sh‐TAK1 group decreased, showing no statistically significant difference compared to the HEI‐OC1 group (Figure 6B,C). Taken together, our data confirmed that inhibiting necroptosis can mitigate the effects of decreased TAK1 expression in HEI‐OC1 cells. This finding may provide a potential therapeutic approach for treating ARHL.

## 4 Discussion

Sensorineural hearing impairment is caused by a wide range of factors, including HCs loss or dysfunction, SGNs degeneration, and SV atrophy (Cui et al. 2020; Marcus et al. 2002; Wiwatpanit et al. 2018). Drug‐induced damage, genetic mutations, and mitochondrial dysfunction can lead to the death of crucial functional cells in the cochlea and cochlear nucleus (Chen et al. 2014; Fu et al. 2022; Qiu et al. 2022; Xu et al. 2023; Zhong et al. 2012). In this process, numerous mechanisms of cell death are involved, with apoptosis (Hong et al. 2023) and autophagy (He et al. 2021) being the most extensively studied. In recent years, as research into cell death has advanced, pyroptosis, ferroptosis, and necroptosis have garnered significant attention (Liu et al. 2024; Wang et al. 2024; Zhou et al. 2023). Necroptosis is the most thoroughly studied form of programmed necroptosis. Different cell death pathways are not isolated; crosstalk and interactions are important to consider when studying programmed cell death pathways.

TAK1 is a key regulator of cell survival and cell death signaling. It can trigger different signal transduction pathways and cellular responses in stressed cells (Sylvain‐Prevost et al. 2015). Under normal conditions, TAK1 can be activated by TNF‐α stimulation, which then combines with IκB kinase, tumor necrosis factor receptor 1 (TNFR1), TNRF1‐associated death domain protein (TRADD), TNF receptor‐associated factors (TRAF) 2 and 5, receptor‐interacting serine/threonine protein kinase 1 (RIPK1), inhibitors of apoptosis proteins 1 and 2, and various types of ubiquitin chains to form a membrane complex I, activating NF‐κB to drive the transcription of survival‐promoting genes (Dondelinger et al. 2013). However, loss of the TAK1 gene through certain microbial or pharmacological routes triggers the assembly of PAN‐optotic cell death complexes, leading to cell death through various mechanisms, including apoptosis, pyroptosis, and necroptosis (Malireddi et al. 2020). When TAK1 is inhibited, membrane complex I separates from TNFR1 to form cytoplasmic protein complex IIa, consisting of TRADD, Fas‐associated death domain (FADD), RIPK1, CASPs 8, 3, and 7, and other caspases, leading to apoptosis (Guo et al. 2016). In addition, complex IIa triggers NOD‐like receptor family pyrin domain containing 3 and CASP8‐mediated gasdermin D cleavage, causing pyroptosis (Orning et al. 2018; Sanjo et al. 2019). Once TAK1 and CASP8 are simultaneously inhibited, complex IIb, including FADD, RIPK1, and RIPK3, will be formed (Yuan, Amin, and Ofengeim 2019) to induce necroptosis by phosphorylating the downstream molecule MLKL (Conos et al. 2017).

During the observation period, DBA/2 J mice displayed early hearing loss, accompanied by morphological changes such as reduced HCs count, decreased SGN density, and narrowed SV. This finding is consistent with previous findings (Willott and Bross 1996). Therefore, DBA/2 J mice serve as a suitable model for ARHL. In this study, qRT‐PCR and immunofluorescence staining revealed relatively high levels of TAK1 expression in cochlear tissue, where it was uniformly distributed in the cytoplasm of cochlear HCs, SGNs, and SV cells. TAK1 gene expression analysis indicated that the TAK1 levels were highest in the cochlea at 4 weeks of age but decreased sharply with age, showing a negative correlation. This showed that the decline in TAK1 expression with age may contribute to cochlear damage, implicating TAK1 loss as a key factor in the pathogenesis of ARHL. In addition, in the absence of TAK1, levels of necroptotic factors *RIPK3* and *MLKL* were significantly elevated. This suggests that reduced TAK1 expression may contribute to cochlear damage and ARHL pathogenesis, with TAK1 potentially mediating necroptosis in this process.

D‐gal can induce oxidative stress and mitochondrial damage in various tissues, contributing to aging (Guo et al. 2020). Previous studies have demonstrated that D‐gal can cause auditory dysfunction, cellular senescence in cochlear tissue, and a reduction in neurofilaments (He et al. 2021; Li et al. 2018). Consequently, D‐gal‐induced HEI‐OC1 cells have become a widely used aging cell model for ARHL. Following treatment with 40 mg/mL D‐gal for 48 h, we successfully established the HEI‐OC1 cell aging model, which exhibited increased ROS levels and significantly elevated expression of aging‐related genes P16 and P21. Compared to normal cells, TAK1 mRNA and protein levels were significantly reduced in the D‐gal‐induced cell aging model. Taken together with the previous experiments, these findings provide strong evidence that TAK1 expression is closely related to cellular aging.

We utilized HEI‐OC1 cells to construct TAK1 knockdown and overexpression models to investigate the regulatory effect of TAK1 expression on cellular function. In the TAK1 knockdown group, necroptosis was observed, accompanied by a significant increase in necroptotic factors *RIPK3* and *MLKL*, and a decrease in cell survival factors *NF‐κB* and *CASP8*. In the TAK1 overexpression group, although *NF‐κB* and *CASP8* remained decreased, *RIPK3* and *MLKL* were further reduced, indicating inhibited cell necroptosis. It is concluded that when TAK1 expression decreases and CASP8 expression is inhibited, cells will shift from survival‐promoting pathways to necroptotic pathways, leading to cell necroptosis. In contrast, necroptosis was reduced in the TAK1 overexpression group. Upon adding the necroptosis inhibitor to TAK1‐knockdown cells, we observed that the cell damage induced by TAK1 knockdown was reversed, and the expression levels of the inflammatory factors IL‐1β and LDH were comparable to the normal group. This further supports the notion that TAK1 mediates cell death by regulating the necroptotic pathway and that TAK1‐mediated necroptosis significantly affects HEI‐OC1 cell function. These findings suggest potential directions for future research. Given that TAK1 is a kinase, excessive regulation of its expression may have deleterious effects on cells. Our results suggest that necroptosis inhibitors may provide a promising alternative for the treatment of ARHL.

Inflammation is a defensive response that helps the body to protect itself from infection and tissue damage (Ekabe et al. 2021; He et al. 2020). Acute inflammation is a physiological response to injury or infection that recruits immune cells to eliminate invading pathogens and reduce tissue damage. However, excessive or persistent inflammation can negatively impact cell survival (Teissier, Boulanger, and Cox 2022). With aging, the body becomes particularly vulnerable to sensorineural hearing loss due to chronic inflammation. In our study, we found that the TAK1 expression level was highly correlated with ABR thresholds and morphological changes in2–16‐week‐old DBA/2 J mice cochleae, with increased expressions of necroptosis factors, particularly at 16 weeks. We further demonstrated that TAK1 significantly mediates necroptosis in auditory HEI‐OC1 cells. Our results revealed that the decreased TAK1 expression caused necroptosis in the cochleae of DBA/2 J mice, which may be an important cause of ARHL. However, a limitation of this study is the lack of a verified TAK1‐necroptosis mechanism in TAK1‐gene overexpressed mice.

## 5 Conclusions

Overall, TAK1 was shown to be a key factor regulating cell survival and death upstream of the inflammatory pathway. This study demonstrated that the TAK1‐mediated necroptosis pathway plays an important role in the pathogenesis of ARHL.

## Author Contributions

Hanjing Wang completed the experiment and wrote the original manuscript. Yayun Lv, He Zhao, and Zhihong Hao assisted with the experiment and collected data. Xiaoyu Zhai and Yan Wang did the statistical analysis. Jingjing Qiu and Liang Chen assisted in figure preparation. Jiamin Zhou and Limei Cui conceived and designed the experiments. Yan Sun reviewed and revised the manuscript. All authors critically reviewed the article and approved the final manuscript.

## Ethics Statement

This study is approved by the Animal Use and Care Committee of the Yantai Yuhuangding Hospital Affiliated with Qingdao University; the ethical approval number was 2022‐377.

## Consent

All authors approved the final manuscript and the submission to this journal.

## Conflicts of Interest

The authors declare no conflicts of interest.

## Supporting information


**Figure S1.** TAK1 decrease and necroptosis increase in D‐gal‐induced aging HEI‐OC1 cells. (A) The cellular viability of HEI‐OC1 cells treated with varying concentrations of D‐gal for 24, 48, and 72 h were shown. (B) The relative mRNA expression of P16 and P21 were detected by qRT‐PCR. (C) The quantitative statistical plots of the relative protein expression of P16 and P21 by immunofluorescence staining. Each data was repeated in three independent assays (**p < 0.01, ***p < 0.001, ****p < 0.0001; ns, no significance).

**Figure S2.** HEI‐OC1 cells were treated with 0, 20, 40, 60, 80, and 100 μM Necrostatin‐1 for 48 h. Cellular viability was then detected by CCK‐8. Each data was repeated in three independent assays.

## Data Availability

The data and materials in this study are reasonably acquired from the corresponding author.
