# Supplementary figures and images for: Transforming Growth Factor‐β‐Activated Protein 1 (TAK1) Regulates Necroptosis in Age‐Related Hearing Loss
Source: Aging Cell. 2025 Feb 28;24(6):e70013. doi: 10.1111/acel.70013 (PMC12151908; doi:10.1111/acel.70013)

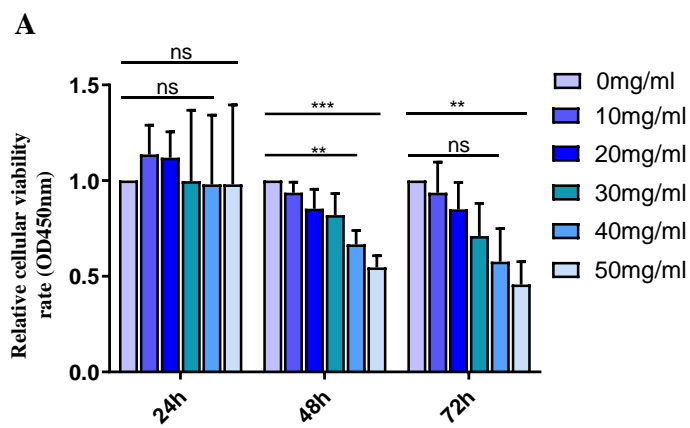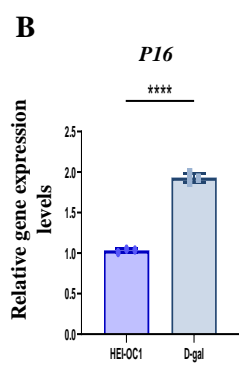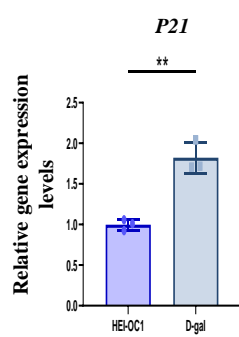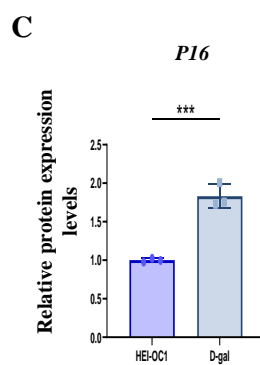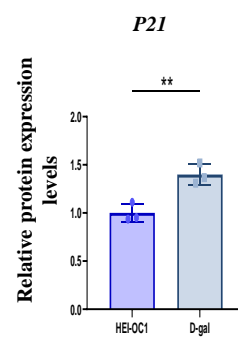

Supplement: Supplementary file 1 — Figure S1. TAK1 decrease and necroptosis increase in D‐gal‐induced aging HEI‐OC1 cells. (A) The cellular viability of HEI‐OC1 cells treated with varying concentrations of D‐gal for 24, 48, and 72 h were shown. (B) The relative mRNA expression of P16 and P21 were detected by qRT‐PCR. (C) The quantitative statistical plots of the relative protein expression of P16 and P21 by immunofluorescence staining. Each data was repeated in three independent assays (**p < 0.01, ***p < 0.001, ****p < 0.0001; ns, no significance). [file ACEL-24-e70013-s001.pdf]

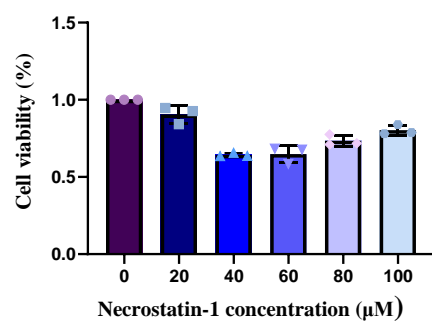

Supplement: Supplementary file 2 — Figure S2. HEI‐OC1 cells were treated with 0, 20, 40, 60, 80, and 100 μM Necrostatin‐1 for 48 h. Cellular viability was then detected by CCK‐8. Each data was repeated in three independent assays. [file ACEL-24-e70013-s002.pdf]
